# The Fer Tyrosine Kinase Mediates EGFR Activation in Sperm Capacitation

**DOI:** 10.3390/ijms262211218

**Published:** 2025-11-20

**Authors:** Odeya Yemini-Talbi, Uri Nir, Haim Breitbart

**Affiliations:** The Mina & Everard Faculty of Life Sciences, Bar-Ilan University, Ramat-Gan 5290002, Israeluri.nir@biu.ac.il (U.N.)

**Keywords:** spermatozoa, capacitation, acrosome, reaction, fer kinase, EGFR

## Abstract

Mammalian sperm cells must undergo several processes collectively called capacitation before carrying out the acrosome reaction (AR), which is required for sperm penetration into the oocyte. The spontaneous acrosomal reaction (SAR), which can occur before the sperm cell reaches the vicinity of the oocyte, impairs the fertilizing ability of the sperm. This study examined the role of the Fer tyrosine kinase in sperm fertilizing activity. Inhibition of the Fer activity led to a 75% reduction in IVF rates in mice, indicating a critical role for Fer in fertilization. Further investigation of Fer’s role during sperm capacitation focused on its potential interaction with the epidermal growth factor receptor (EGFR). Inhibition of Fer during capacitation significantly decreased the EGFR activation state and increased the incidence of SAR, whereas inhibition of Fer during the acrosome reaction step had no effect on the EGF-induced AR. The effects of Fer inhibition on EGFR activation and SAR enhancement are mediated by the Ca^2+^ channel, CatSper. Notably, reduction in Ca^2+^ influx by CatSper inhibition revealed a significant increase in Fer phosphorylation/activation, while increasing intracellular Ca^2+^ concentrations completely inhibited this effect. Additionally, we show that Fer activation depends on a signaling cascade involving protein kinase A (PKA) that leads to EGFR activation through the following pathway: HCO_3_^−^ → SAC → cAMP → PKA → Src → Fer → EGFR. Collectively, we decipher in this work a new regulatory cascade that leads to the Fer-directed activation of EGFR in sperm capacitation.

## 1. Introduction

Prior to fertilization, mammalian spermatozoa must undergo a series of biochemical processes to acquire the capacity to fertilize the egg. This process is called capacitation and it is necessary to occur in the sperm in order bind to the Zona Pellucida (ZP) of the oocyte. As a result of this binding, the spermatozoon undergoes the physiological acrosome reaction (AR), a process that allows penetration and fertilization of the egg (reviewed in [1]).

However, spermatozoa can undergo so-called spontaneous acrosome reaction (sAR) before reaching the egg’s surroundings, a process that restricts successful fertilization. Sperm adopt several mechanisms and regulatory cascades to protect from the onset of sAR. These pathways include phospholipase D (PLD) and calmodulin-kinase II (CaMKII) [2], phosphatidyl-unositol-3-kinase (PI3K) and Ezrin (Rev.in [3]) and pH-dependent Ca^2+^ oscillations [4,5,6]. All of these pathways modulate actin polymerization (F-actin formation) during sperm capacitation.

The formation of F-actin in the sperm tail is important for the development of hyperactivated motility (HAM) (Rev.in [3]) in capacitated sperm and F-actin expressed in the sperm head is essential for prevention of spontaneous AR (SAR) (Rev.in [3]). Our lab suggested that during sperm capacitation, premature interaction between the outer acrosomal and the overlaying plasma membranes is inhibited by the F-actin network within the sperm head, which forms a physic-mechanical barrier that prevents sAR (Rev.in [3]).

We have shown that phospholipase D (PLD) mediates F-actin formation in sperm cells (Rev.in [3]). Phosphatidic-acid (PA), the product of phosphatidyl-choline hydrolysis by PLD, is essential for the tyrosine-kinase Fer activation [7]. Indeed, we showed that inhibition of Fer abrogates F-actin formation during human sperm capacitation [8], and that Src-family tyrosine kinases mediate Fer phosphorylation and activation in human sperm cells [8]. Sperm contain epidermal growth factor receptor (EGFR) which participates in the AR and is activated by Src (Rev.in [3]). Thus, we hypothesized that EGFR might be involved in the mechanism through which Fer promotes sperm capacitation. In this study, we show that Fer activates the EGFR, resulting in a proper completion of sperm capacitation.

## 2. Results

### 2.1. Fer Inhibition Enhances Spontaneous but Not Induced Acrosomal Reaction

Previous studies in our laboratory found that inhibition of Fer at the beginning of the capacitation process by the selective Fer inhibitor—E260 [9]—leads to an increase in the percentage of human sperm cells that undergo spontaneous acrosomal reaction—SAR [8]. We adopted the Fer inhibitor—E260—that was shown by us [9,10], and by others [11,12], to specifically inhibit this kinase.

**Figure 1:**
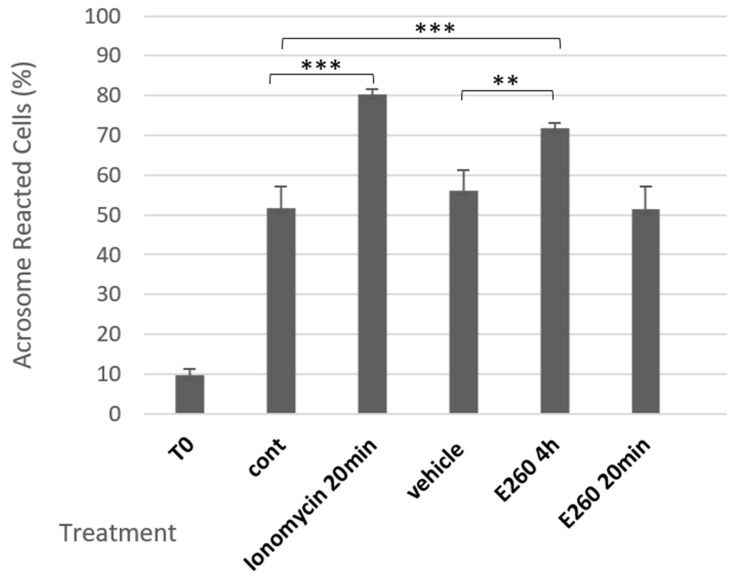
**Effect of Fer inhibition on SAR in bovine spermatozoa.** Frozen bovine spermatozoa at a concentration of 1 × 10^8^ cells/mL were thawed and incubated in capacitation medium (mTALP) for 4 h in the absence (cont) or presence of the inhibitor E260 [20 µM], or its solvent vehicle. An additional group of cells was incubated for 3 h 40 min without treatment, followed by 20 min incubation with E260. As a positive control, the acrosome reaction (AR) was induced at the end of incubation by adding the calcium ionophore, ionomycin, at 10 µM. T0 represents the AR rate at zero time of incubation). Cells were fixed on slides, stained, and analyzed as described in the Methods section. The values represent the mean ± standard deviation of duplicates from three experiments. ** *p* < 0.01, and *** *p* < 0.001, significant differences compared to the corresponding control.

Here we extend these results and show that treatment of bovine sperm with E260 added at zero time before starting capacitation and during the capacitation time caused significant increase in the spontaneous AR (SAR) but has no effect on induced AR (iAR) level when this inhibitor was added after capacitation (Figure 1). Thus, Fer activity ensures completion of the capacitation process preventing SAR, rather than directly modulating the acrosome reaction process.

### 2.2. Involvement of Calcium Channels in the Spontaneous Acrosomal Reaction Induced by Fer Inhibition

**Figure 2:**
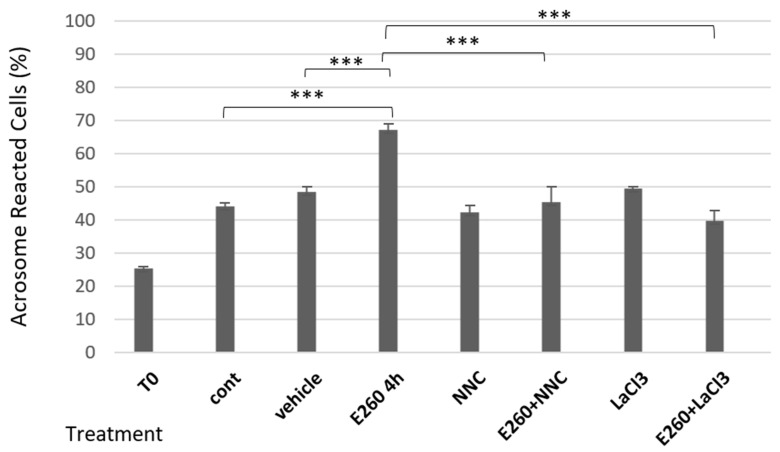
**Effect of calcium channel inhibition on SAR caused by E260.** Frozen bovine spermatozoa at a concentration of 1 × 10^8^ cells/mL were thawed and incubated in capacitation medium (mTALP) for 15 min in the absence (con) or presence of the inhibitor E260 [20 µM] and its solvent vehicle. Some samples were further incubated with the calcium channel blockers NNC-55-0396 (5 µM) or LaCl_3_ (dissolved in H_2_O) (0.5 mM) for an additional 3 h 45 min. T0 is the AR rate at zero time of incubation. Cells were fixed on slides, stained, and analyzed as described in the Methods”. The values represent the mean ± standard deviation of duplicates from three experiments. *** *p* < 0.001, significant differences compared to the corresponding control.

**Figure 3:**
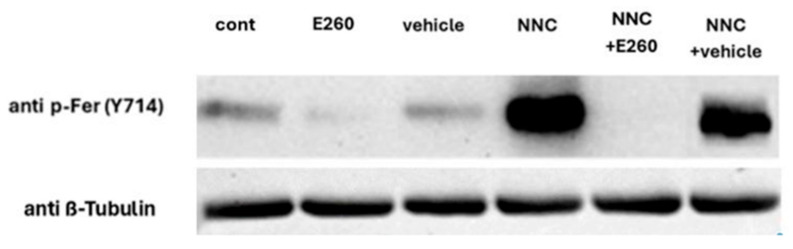
**Effect of CatSper channel inhibition on Fer phosphorylation.** Frozen bovine spermatozoa at a concentration of 1 × 10^8^ cells/mL were thawed and incubated in capacitation medium (mTALP) for 15 min in the absence (cont) or presence of the inhibitor, E260 [20 µM] and its solvent vehicle. Subsequently, cells were incubated for an additional 45 min in the absence or presence of NNC-55-0396 (5 µM). Calyculin A (0.1 mM), a phosphatase inhibitor, was added 25 min after the start of incubation. At the end of the incubation, proteins were extracted and analyzed by Western blot using the following antibodies: anti-phospho-Fer (Tyr714) upper panel, and anti-β-tubulin (loading control) lower panel.

The acrosomal reaction is induced in part by an increase in intracellular calcium concentration, which promotes fusion of the membranes. One of the main channels that controls the intracellular calcium concentration in sperm cells is the sperm-specific Ca^2+^ channel, CatSper [13]. We next tested the effect of Ca^2+^ channel activity on the onset of SAR upon Fer inhibition. As shown in Figure 2, the enhancing effect of E260 added at zero time before starting capacitation on SAR is completely inhibited by the CatSper inhibitor NNC-55-0396, indicating that activation of CatSper is a main factor mediating the increase in SAR in response to Fer inhibition. The inhibition of E260-induced SAR by La^3+^, which inhibits all Ca^2+^ channels, further supports this conclusion. The specificity of the CatSper inhibitor as Ca^2+^ influx inhibitor is seen in Figure 3 in which NNC-55-0396 enhances Fer phosphorylation/activation, indicating that the reduction in SAR by this inhibitor is due to blocking Ca^2+^ influx and not through possible inhibition of the Fer activity.

### 2.3. CatSper Inhibition Enhances Fer Phosphorylation/Activation

**Figure 4:**
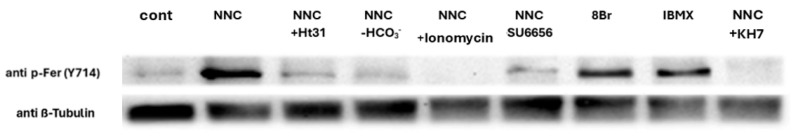
**Effect of various PKA pathway modulators on Fer phosphorylation.** Frozen bovine spermatozoa at a concentration of 1 × 10^8^ cells/mL were thawed, and starved for 2 h in NKM medium, followed by incubation in capacitation medium (mTALP) with or without bicarbonate (NNC–HCO_3_^−^) for 15 min in the absence (con) or presence of the following compounds: Ht31 (5 µM), SU6656 (50 µM), KH7 (10 µM), 8Br-cAMP (1 mM), or IBMX (1 mM). Cells were then incubated for an additional 45 min in the absence or presence of NNC-55-0396 (5 µM). For the NNC + ionomycin condition, cells were incubated with both compounds from the beginning of the incubation. Calyculin A (0.1 mM), a phosphatase inhibitor, was added 25 min after the start of incubation. At the end of the incubation, proteins were extracted and analyzed by Western blot using the following antibodies: anti-phospho-Fer (Tyr714) and anti-β-tubulin (loading control).

Since Fer inhibition caused an increase in SAR, and inhibition of Ca^2+^ transport into the cells reduced this effect, we asked whether inhibition of Ca^2+^ entry via CatSper would affect the Fer phosphorylation/activation level. As shown in Figure 3, (see full blot in Original Western Blotting image 3S) inhibition of CatSper by NNC resulted in a significant increase in Fer kinase phosphorylation compared to the control, and this effect was blocked by E260 added at zero time before starting capacitation. These results suggest that low concentrations of intracellular Ca^2+^ increase Fer activity. Accordingly, increasing the [Ca^2+^]_i_ level using the Ca^2+^-ionophore ionomycin caused complete inhibition of the enhancing effect of NNC on the Fer activation state (p-Fer) (Figure 4 and Original Western Blotting image 4S). Thus, we conclude that intracellular Ca^2+^ concentration regulates Fer activation.

**Figure 5:**
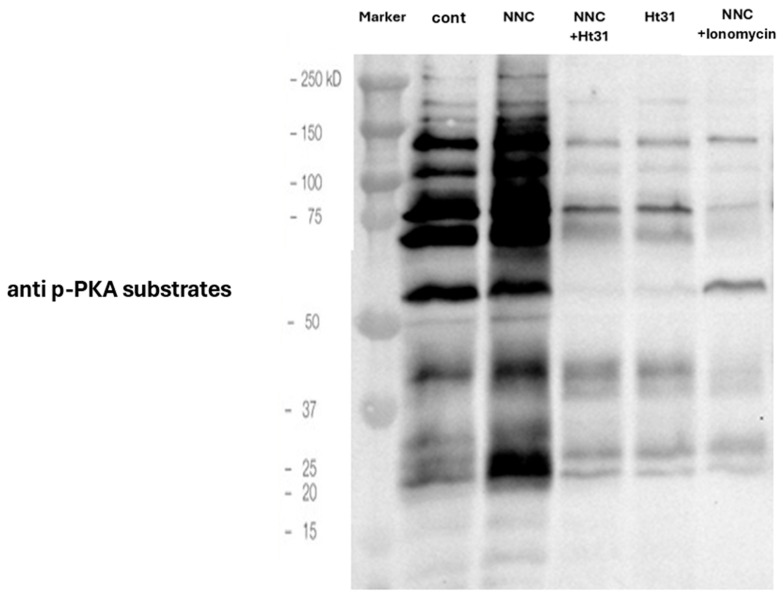
**Effect of NNC on phosphorylation of PKA substrates.** Frozen bovine spermatozoa at a concentration of 1 × 10^8^ cells/mL were thawed and starved for 2 h in NKM medium, followed by incubation in capacitation medium (mTALP) for 15 min in the absence (con) or presence of Ht31 (5 µM) or ionomycin (10 µM). Cells were then incubated for an additional 45 min in the absence or presence of NNC-55-0396 (5 µM). For the NNC + ionomycin condition, cells were incubated with both compounds from the start of the incubation. Calyculin A (0.1 mM), a phosphatase inhibitor, was added 25 min after the start of incubation. At the end of the incubation, proteins were extracted and analyzed by Western blot using anti-p-PKA substrate antibodies.

In a previous study in our laboratory, it was found that protein kinase A (PKA) mediates the induced phosphorylation of Fer in human sperm, via activation of Src [8]. To extend this finding, we tested the effect of elevated level of the PKA activator-cAMP on bovine sperm by adding the cell permeable analog of cAMP-8Br-cAMP, or by inhibiting cAMP-phosphodiesterase using IBMX. Elevation of intracellular cAMP caused a significant increase in p-Fer level (Figure 4), indicating that PKA mediates Fer phosphorylation in bovine sperm as well. Moreover, Fer phosphorylation boosted by the CatSper inhibitor NNC was inhibited in the presence of various substances that reduce PKA activity: Ht31 (a PKA-specific inhibitor), absence of bicarbonate [activator of soluble adenylyl cyclase (SAC)], and KH7 (inhibitor of SAC) (Figure 4). In addition, inhibition of Src kinase by SU6656 caused significant reduction in p-Fer (Figure 4). These results indicate that the upregulating effect of NNC on the p-Fer level is PKA/Src-dependent, and is caused by reducing the concentration of calcium ions in the cell. Moreover, as shown in Figure 5, NNC causes an increase in the phosphorylation of PKA substrates, and this effect is inhibited by the PKA inhibitor HT31 or by increasing [Ca^2+^] I using ionomycin. Thus, inhibition of CatSper by NNC stimulates PKA activity, leading to Fer activation in bovine sperm.

### 2.4. Studying the Regulatory Role of Fer in EGFR Activity

Previous studies have shown that Src mediates EGFR activation in bovine sperm [14], and we showed here that Src mediates Fer activation, as well (Figure 4). These results raised the hypothesis that there is a functional relationship between Fer and EGFR, since both are affected by the activity of the Src tyrosine kinase.

**Figure 6:**
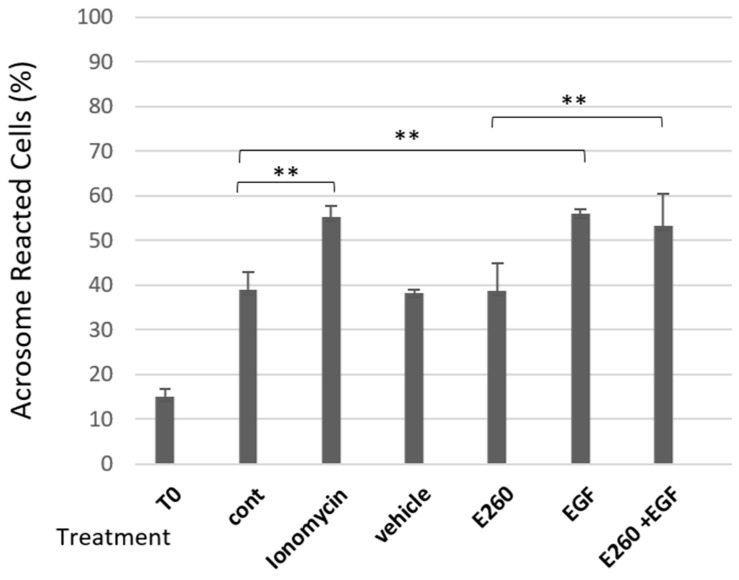
**Effect of Fer inhibition on induced AR (iAR) caused by EGF**. Frozen bovine spermatozoa at a concentration of 1 × 10^8^ cells/mL were thawed and incubated in capacitation medium (mTALP) for 3 h 45 min (cont). Then, an aliquot of cells was incubated for 15 min with the inhibitor E260 [20 µM], or its solvent vehicle. EGF (5 ng/mL) was then added to the cells for an additional 15 min. As a positive control, the acrosome reaction (AR) was induced at the end of incubation by adding the calcium ionophore, ionomycin (10 µM). T0 represents the rate of AR at zero time. Cells were fixed on slides, stained, and analyzed as described in the Methods. The values represent the mean ± standard deviation of duplicates from three experiments. ** *p* < 0.001, significant difference compared to the corresponding control.

**Figure 7:**
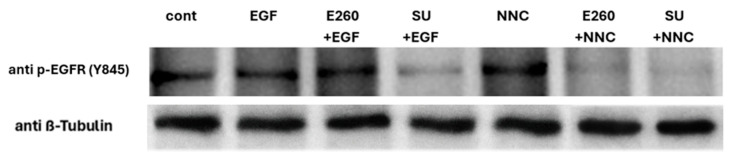
**Effect of Fer and CatSper inhibition on EGFR phosphorylation.** Frozen bovine spermatozoa at a concentration of 1 × 10^8^ cells/mL were thawed and starved for 2 h in NKM medium, followed by incubation in capacitation medium (mTALP) with Calyculin A (0.1 mM), a phosphatase inhibitor, for 5 min. Cells were then incubated for an additional 15 min in the absence (cont) or presence of the inhibitors SU6656 (50 µM), E260 (20 µM). EGF (5 ng/mL) and NNC-55-0396 (5 µM) were subsequently added to the cells for an additional 5 min. At the end of the incubation, proteins were extracted and analyzed by Western blot using anti-p-EGFR (Tyr845) and anti-β-tubulin antibodies.

**Figure 8:**
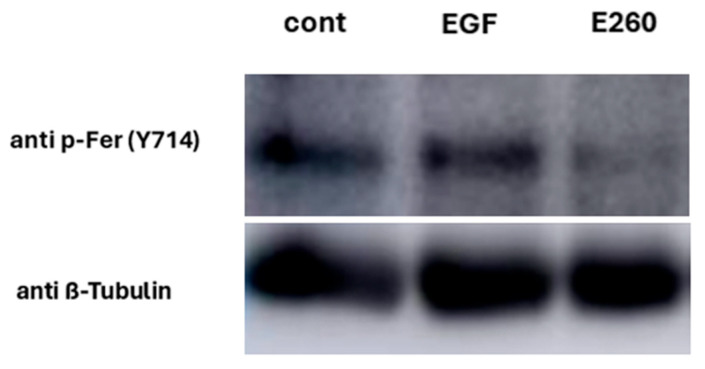
**Effect of enhanced EGFR activity on Fer phosphorylation.** Frozen bovine spermatozoa at a concentration of 1 × 10^8^ cells/mL were thawed and incubated in capacitation medium (mTALP) for 15 min in the absence or presence of the inhibitor E260 [20 µM], followed by an additional 45 min in the absence or presence of EGF (5 ng/mL). Calyculin A (0.1 mM), a phosphatase inhibitor, was added 25 min after the start of incubation. At the end of the incubation, proteins were extracted and analyzed by Western blot using the following antibodies: anti-phospho-Fer (Tyr714) and anti-β-tubulin.

Bovine sperm contain EGFR, and its activation by EGF in capacitated sperm caused a significant increase in the AR level [15]. We showed above (Figure 1) that Fer ensures correct completion of the sperm capacitation, but it is not involved in the regulated AR process. This suggests that Fer is not a downstream effector of the EGF-driven activation of EGFR. Accordingly, activation of EGFR by adding its ligand EGF to capacitated sperm caused an increase (43%) in AR level, and this effect was not affected by Fer inhibitor E260 added at the end of the capacitation time (Figure 6), further indicating that the EGF-induced AR is not mediated by Fer. This result supports our findings that Fer is not directly involved in the acrosomal reaction process. Moreover, this result suggests that Fer is regulatory-wise localized upstream to EGFR, since direct activation of EGFR by EGF is not affected by Fer inhibition (Figure 7). Also, if EGFR is localized upstream to Fer, it is expected to see that activation of EGFR by EGF would upregulate p-Fer, but this is not the case (Figure 8 and Original Western Blotting image 8S), further corroborating the regulatory localization of Fer upstream to EGFR.

**Figure 9:**
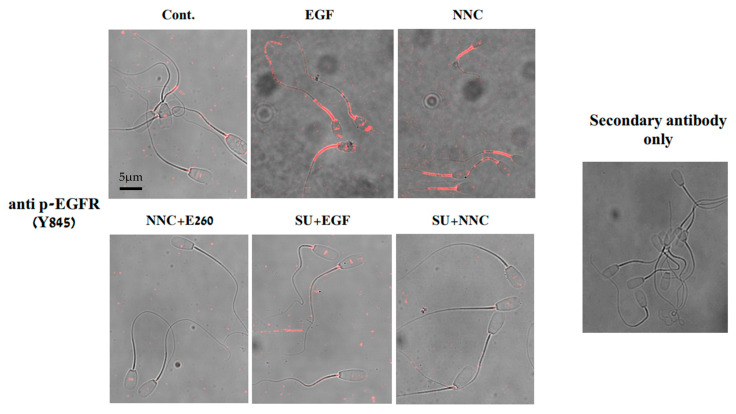
**Effect of CatSper inhibition on EGFR phosphorylation.** Frozen bovine spermatozoa at a concentration of 1 × 10^8^ cells/mL were thawed and starved for 2 h in NKM medium, followed by incubation in capacitation medium (mTALP) for 5 min with Calyculin A (0.1 mM), a phosphatase inhibitor. Cells were then incubated for 15 min without treatment (control) or in the presence of the inhibitors SU6656 (50 µM) and E260 (20 µM). EGF (5 ng/mL) and NNC-55-0396 (5 µM) were subsequently added for an additional 5 min. Cells were stained with anti-p-EGFR (Tyr845) antibody as described in the Methods. As a control, staining was performed on cells treated with EGF and secondary antibody only. Representative image of bovine spermatozoa (×1000). Scale bar = 5 µm.

We showed (Figure 4) that PKA and Src mediate Fer phosphorylation/activation, and previously demonstrated that PKA/Src mediate EGFR activation in bovine sperm [15]. In order to further demonstrate that Fer is localized upstream to EGFR in the pathway, we followed EGFR phosphorylation on tyrosine 845 using Western blotting analysis and immunocytochemical staining. As noted above, the effect of EGF on EGFR phosphorylation was not altered by the Fer inhibitor E260 added before starting capacitation (Figure 7 and Original Western Blotting image 7S), confirming that EGFR is localized downstream to Fer. Moreover, the CatSper inhibitor NNC, which caused a significant increase in p-Fer (Figure 3), also stimulates EGFR phosphorylation, and this effect of NNC is inhibited by E260 or by the Src inhibitor, SU (Figure 7 and Figure 9). These data further corroborate the notion that Fer mediates the phosphorylation/activation of EGFR. Src was shown to phosphorylate EGFR on Tyr 845; the ability of the Src inhibitor, SU, to inhibit EGFR phosphorylation in the presence of EGF or NNC (Figure 7 and Figure 9) indicates the specificity of the test, and the different localizations of Src and Fer in the EGFR-activating cascade.

Immunocytochemical staining of sperm revealed that EGF-stimulated p-EGFR localized to the midpiece and the sperm head, whereas NNC stimulated this phosphorylation in the midpiece only (Figure 9), where Fer is primarily located [16]. Accordingly, the NNC-driven phosphorylation of EGFR in the midpiece was completely eliminated by the Fer inhibitor E260 added before starting capacitation. (Figure 9 lower panel), conclusively showing that the calcium-regulated activation of Fer regulates also the phosphorylation/activation state of EGFR. Quantitative assessment showed that approximately 82% of spermatozoa exhibited p-EGFR staining in both the head and midpiece regions, whereas the remaining cells displayed labeling restricted to the midpiece only. This point is further discussed in the Discussion.

### 2.5. Inhibition of Fer Reduces IVF Rate

Finally, the effect of Fer inhibition on the sperm fertilizing capacity was examined using a mouse in vitro fertilization system. Due to technical constraints, we could not perform the IVF assay in bovine system. Mouse sperm show high similarity with bovine sperm especially in the mechanism that mediates sperm capacitation. Additionally, Fer mediates protein tyrosine phosphorylation in mouse sperm [17], a process that is essential for achieving capacitation in mouse and bovine sperm. Thus, by adopting the mouse IVF system we examined the physiological role of Fer in fertilization. The cleavage rate of oocytes was 80% in the control group, 88% in the sperm cohort treated with vehicle, and 22% in the presence of the Fer inhibitor E260 added to the sperm for 1 h before mixing with the eggs (Table 1). Hence, targeting of Fer causes 72% reduction in the IVF rate, demonstrating the importance of Fer in the fertilization process.

## 3. Discussion

Mammalian sperm are unable to fertilize an egg immediately after ejaculation. To enable fertilization, they must undergo a number of biochemical and morphological processes in the female reproductive system, collectively called capacitation. Only a cell that has undergone this process can interact with the oocyte and undergo the acrosomal reaction required for the sperm cell to penetrate the egg (Rev.in [1]). Spontaneous AR that occurs before the sperm cell reaches the egg region and completes the capacitation process impairs the ability of sperm cell to fertilize [18]. This study examined the role of the Fer kinase in ensuring completion of the capacitation process in bovine sperm cells, focusing on the possible connection with the EGFR. Sperm capacitation is accompanied by a rapid increase in bicarbonate influx which activates soluble adenylyl cyclase and cAMP production [19], leading to PKA activation and an indirect increase in protein tyrosine phosphorylation [20]. PKA is a serine/threonine kinase, lacking tyrosine phosphorylation activity; it was therefore suggested that Fer is responsible for the capacitation-associated protein tyrosine phosphorylation in murine sperm [17]. This suggestion was further supported by our group in human sperm [8], and here in bovine sperm. Using a selective inhibitor for Fer, E260, we found that inhibiting Fer activity during the capacitation process caused a significant increase in the proportion of cells that underwent SAR (Figure 1). In contrast, the inhibition of Fer after the end of the capacitation period did not significantly affect the proportion of these cells that had undergone AR. Moreover, the EGF-induced AR in capacitated cells was not affected by Fer inhibition (Figure 6). These results suggest that Fer activity during the capacitation phase, but not in the acrosome reaction step, is essential for the proper timing, regulation, and completion of the capacitation process, without an interrupting onset of AR.

The involvement of Fer in sperm capacitation is supported by our previous study in which we show that three markers of capacitation, protein tyrosine phosphorylation, hyperactivated motility, and actin polymerization, are significantly inhibited following Fer inhibition [8]. In addition to Fer, sperm contain several mechanisms that protect it from SAR, including pathways mediated via PLD and CaMKII, PI3K Ezrin (Rev.in [3]).

Fer inhibition caused significant increase in SAR (Figure 1) and Fer mediates actin polymerization in bovine sperm [8]. Thus, the enhanced effect of Fer inhibition on SAR is likely to occur due to the inhibition of actin polymerization. We also showed that inhibition of Fer significantly reduced the phosphorylation/activation of cortactin [8]. It has been previously demonstrated that mice devoid of the Fer kinase activity display a reduced level of tyrosine-phosphorylated cortactin [21]. Furthermore, in somatic cells, it was shown that cortactin binds the SH2 sequence of Fer, and that Fer kinase activity is required for cortactin tyrosine phosphorylation, indicating that cortactin is a direct substrate of Fer [22].

Cortactin itself was found to interact with the Arp2/3 complex [23,24], which mediates actin filament branching and F-actin formation [25,26,27]. In addition, cortactin is a stabilizer of actin branches formed by Arp2/3 [24]. Based on these findings, we previously suggested that phosphorylation and activation of cortactin by Fer directs actin polymerization during sperm capacitation [8]. Importantly, we also demonstrated that this effect of Fer is essential for protecting sperm from SAR, thereby enabling the proper completion of the capacitation process.

The increase in SAR caused by inhibition of Fer activity might be a physiological phenomenon related to the concentration of calcium ions in the cell, or an artificial phenomenon indicating cell deformation. To distinguish between these possibilities, we examined how blocking calcium channels, and, in particular, the CatSper channel, affects this increase in SAR. It was found that CatSper inhibition completely blocked the rise in SAR caused by Fer inhibition (Figure 2). This result indicates that the CatSper is a major Ca^2+^ channel that mediates the spontaneous acrosomal reaction caused by Fer inhibition. Moreover, we show that inhibition of CatSper caused a significant PKA-dependent increase in Fer phosphorylation/activation (Figure 3), and this effect is completely eliminated by increasing intracellular Ca^2+^ concentration using Ca^2+^ ionophore (Figure 5). These findings lead to the conclusion that Fer activity is affected by intracellular calcium concentrations, as high calcium concentrations inhibit its phosphorylation, while low calcium increases its phosphorylation. In addition, these observations raise the possibility that a calcium-dependent phosphatase such as calcineurin, which is present in sperm cells [28], is involved in the control of the Fer phosphorylation. Overall, it can be concluded that the CatSper-dependent increase in SAR caused by the inhibition of Fer is due to an increase in the level of Ca^2+^, which is required for the AR.

We have shown that PKA and Src mediate the phosphorylation/activation of Fer in human sperm cells [8]. Here we followed the p-Fer levels using inhibitors or activators of this pathway. Inhibition of sAC, PKA, or Src prevent the elevation of p-Fer caused by CatSper inhibition (Figure 5). Moreover, increasing intracellular cAMP by inhibiting cAMP-phosphodiesterase or by providing 8Br-cAMP to the cells increase the p-Fer levels (Figure 5). Moreover, Fer as well as PKA activities are regulated by intracellular Ca^2+^ concentrations in which at low [Ca^2+^_i_] Fer and PKA are activated, whereas high [Ca^2+^_i_] caused their inhibition (Figure 6). PKA leads to indirect activation of tyrosine kinase, PLD, and CaMKII, three enzymes involved in F-actin formation during capacitation, and protect sperm from SAR (Rev.in [3]). These data further support our notion regarding the modulation of sperm capacitation by Fer activity.

EGFR is partially activated during sperm capacitation by PKA, even in the absence of EGF, resulting in phospholipase D (PLD) activation and actin polymerization [29]. Here, we show that inhibition of Fer by E260 caused significant reduction in p-EGFR induced by NNC (Figure 7 and Figure 9). Moreover, inhibition of Fer did not affect the level of p-EGFR in the presence of EGF (Figure 7) and EGF did not affect p-Fer level (Figure 8). Thus, Fer mediates EGFR phosphorylation independently of EGF and is regulatorily localized upstream to EGFR. Inhibition of Src by SU also caused significant reduction in p-EGFR level induced by NNC, coinciding with Fer inhibition under these conditions (see Figure 4). Our results show that Src has a dual effect in sperm: it mediates indirect p-EGFR via Fer phosphorylation, and in addition directly affects EGFR phosphorylation.

Immunocytochemical staining revealed that EGF stimulates p-EGFR, localized to the midpiece and the sperm head, whereas NNC stimulated this phosphorylation in the midpiece only (Figure 9), where CatSper and Fer are located [16]. This suggests that Fer mediates EGFR phosphorylation in the midpiece but not in the sperm head. This observation is consistent with the fact that the midpiece region is rich in mitochondria, and Fer was found associated with complex I of the electron transport chain in cancer and sperm cells [16].

The strong reduction in IVF rate by Fer inhibition demonstrates the importance of Fer in sperm function. Although Fer^DR/DR^ mice are fertile in vivo [21], they are sub-fertile when fertilized in vitro [17], as we describe here.

In conclusion, we show here that the tyrosine kinase, Fer, modulates sperm capacitation via Src-dependent EGFR activation. Activation of Fer is mediated by PKA/Src, and the level of phosphorylated/activated Fer is regulated by intracellular Ca^2+^ concentrations in which high [Ca^2+^] causes its dephosphorylation and inactivation. Fer activity during sperm capacitation is essential to prevent spontaneous acrosome reaction, which jeopardizes the ability of sperm to fertilize the egg, and therefore, Fer activation enhances the potential of the sperm to achieve fertilization.

## 4. Materials and Methods

### 4.1. Materials

Ionomycin, SU6656, Calyculin A, and protease inhibitor cocktail III were purchased from Calbiochem (San Diego, CA, USA); St-HT31 peptide was purchased from Promega (Madison, WI, USA); anti-Fer SH2 (produced by our laboratory) was used at 1:500. Anti PKA substrate (9624, Cell Signaling, Danvers, MA, USA 1:1000), anti-phospho-EGFR tyrosine 845 (2231S, Cell Signaling) was used at 1:1000 for WB and 1:50 for IC. Secondary anti-Rabbit and Mouse IgGHRP (111-035-144 and 115-035-062 Jackson ImmunoResearch, West Grove, PA, USA), 1:10,000.

Luminata TM Forte Western HRP Substrate was purchased from Millipore (Billerica, MA, USA). All other chemicals were purchased from Sigma-Aldrich (Rehovot, Israel), unless otherwise stated.

The Fer inhibitor, E260 [Synthesized by ChemDiv. Inc., San Diego, CA, USA], was dissolved and incorporated in chromophore-based vesicles comprising 10% chromophore suspended in phosphate-buffered saline, containing 10% ethanol, to achieve a final concentration of 10 mM, as described [9].

### 4.2. Sperm Preparation

Frozen bovine sperm capsules obtained from “ZION” Israeli Breeding and Artificial Insemination Company Ltd. were thawed in NKM buffer (110 mM NaCl, 5 mM KCl, 20 mM 3-N-morpholinoprpanesulfonic acid (MOPS) (pH 7.4)), at 37 °C. The sperm were washed three times by centrifugation (780× *g* for 10 min at room temperature) in NKM and allowed to swim up. Washed cells were counted and maintained at 37 °C until use.

Sperm capacitation: Sperm pellets were resuspended to a final concentration of 1 × 10^8^ cells in mTALP (Modified Tyrode solution) medium (100 mM NaCl, 3.1 mM KCl, 1.5 mM MgCL_2_, 0.92 mM KH_2_PO_4_, 25 mM NaHCO_3_, 20 mM Hepes (pH 7.4), 0.1 mM sodium pyruvate, 21.6 mM sodium lactate, 10 IU/mL penicillin, 1 mg/mL bovine serum albumin (BSA), 20 mg/mL heparin, and 2 mM CaCl_2_). The samples were then incubated at 38 °C for 4 h in a shaker bath [15]. The capacitation state of the sperm was confirmed by examining their ability to undergo an induced acrosome reaction by addition of the calcium ionophore, ionomycin, at the end of the capacitation time.

### 4.3. Assessment of Acrosome Reaction

The percentage of acrosome-reacted sperm was determined microscopically on air-dried sperm smears using fluorescein isothiocyanate (FITC)-conjugated Pisum sativum agglutinin (PSA) [15]. There is limitation of this assay since membrane damage would give a positive result, especially when SAR is determined. However, the fact that inhibition of Ca^2+^ channels causes significant decrease in SAR provides strong evidence that no significant membrane damage occurs. An aliquot of sperm was smeared on a glass slide and allowed to air dry. The sperm were then fixed in methanol for 20 min at room temperature, washed three times at 5 min intervals with TBS, air-dried, and incubated with FITC-PSA (60 mg/mL in TBS) for 35 min, washed twice with water at 5 min intervals, air-dried, and mounted with ProLong Gold Antifade (Thermo Fisher Scientific Inc., Waltham, MA, USA). The slides were then examined for acrosome-reacted sperm using fluorescence microscopy. At least 200 cells per slide, on duplicate slides (total 400 cells), were examined per experiment. Cells with PSA staining at the acrosome region were considered acrosome-intact; those with equatorial staining or no staining were considered acrosome-reacted.

### 4.4. Immunoblot Analysis

Sperm were washed by centrifugation for 5 min at 10,000× *g*, 4 °C. The pellet was resuspended in Tris-buffered saline (TBS), pH 7.6, and centrifuged again to remove excess BSA. Lysis buffer (50 mM Tris_HCl pH 7.5, 150 mM NaCl, 6% sodium dodecyl sulfate (SDS), protease inhibitor cocktail (1:100), 50 mM NaF, 50 mM pyrophosphate, 0.2 mM Na_3_VO_4_, and freshly added 1 mM phenylmethylsulfonyl fluoride (PMSF)) was added to the pellet [15]. The lysate was vortexed for 15 min at room temperature and then centrifuged for 5 min at 10,000× *g* at room temperature. The supernatant was transferred to new tubes, and the protein concentration was determined by the Bradford method [30] or by the bicinchoninic acid method [31]. Concentrated sample buffer was added to the supernatant and boiled for 5 min. Prepared lysates were separated on 10% SDS polyacrylamide gels, and then electrophoretically transferred to nitrocellulose membranes at 300 mA for 1 h.

Blots were stained with Ponceau solution to confirm equal loading and even transfer. The blots were then blocked for 30 min at room temperature with 1% BSA in TBS, pH 7.6, containing 0.1% Tween 20 (TBST). The membranes were incubated overnight at 4 °C with primary antibodies diluted in 1% BSA in TBST. The membranes were washed three times with TBST, and then incubated for 1 h at room temperature with specific HRP-linked secondary antibodies diluted 1:5000 in TBST and 1% BSA. The membranes were washed again three times with TBST, and visualized by enhanced chemoluminescence reagent.

### 4.5. IVF Determination

Female (C57BlxA.G) mice 6–8 weeks old were super-ovulated with 5 IU of PMSG (Pregnant Mare’s Serum Gonadotropin) followed at a 48 h interval by 5 IU of hCG (Human Chorionic Gonadotropin), and sacrificed between 12 and 17 h after the hCG injection. Oocytes were liberated from the ampullae into M16 medium. Epididymal sperm from one mouse (1 × 10^7^ cells/mL) were prepared and capacitated as described. Sperm were added at 1 × 10^6^ cells/mL and were incubated in 100 μL droplet (on average 15–25 oocytes were present in each droplet for each experiment) and incubated at 37 °C in 5% CO_2_ for 24 h. Then, the oocytes were examined under ×50 magnification of the dissecting microscope to determine the number of 1-cell and 2-cell embryos present [32].

### 4.6. Immunocytochemical Staining

Sperm cells at a concentration of 1 × 10^8^ cells/mL were incubated for 20 min and subjected to various treatments. Sperm samples (10 µL) were taken from and smeared onto glass slides, which were then air-dried. Following drying, the slides were fixed in a washing chamber containing 2.5% formaldehyde in TBS for 10 min at room temperature. After drying, cells were permeabilized for 30 min with 0.5% Triton X-100 in TBS, followed by three washes with TBS for 5 min each.

Immunocytochemistry was performed following the protocol described by de Oliveira et al. [33], with modifications optimized for detecting FER and EGFR in bovine spermatozoa. Briefly, sperm smears were fixed in 4% paraformaldehyde for 10 min, permeabilized with 0.5% Triton X-100 for 30 min, and blocked with 5% BSA in TBS for 30 min at room temperature. Samples were incubated with primary antibodies against FER or EGFR (1:50 in TBS-T containing 1% BSA) for 2.5 h at 37 °C, followed by incubation with fluorophore-conjugated secondary antibodies (1:50, 2 h at 37 °C in the dark). After washing, slides were air-dried and mounted with antifade medium (VECTAshield^®^ Vector Lab., Newark, CA, USA). Negative controls were performed without primary antibodies. Slides were examined under a Zeiss fluorescence microscope at 1000× magnification.

The cells were imaged under a Leica Stellaris confocal fluorescence microscope at 60× magnification.

### 4.7. Statistical Analysis

Data are expressed as mean ± standard deviation of the normalized values and homogeneity of variance of at least three experiments for every determination. A one-way analysis of variance (ANOVA) was performed to compare mean sperm cell counts among the different treatment groups. When a significant main effect was detected, Tukey’s post hoc test was applied for pairwise comparisons. Normality of residuals was assessed using the Shapiro–Wilk test, and homogeneity of variances was verified using Levene’s test. Significance level of *p* < 0.05 was considered statistically significant.

## Figures and Tables

**Table 1:** **Effect of Fer inhibition on fertilization rates in mice.** Mouse spermatozoa were incubated in capacitation medium for 1 h in the presence or absence of E260 [20 µM] or its solvent vehicle. Following incubation, IVF was performed with these cells as described in the Methods section, and fertilization success was determined for each treatment. The values represent the mean ± standard deviation of duplicates from three experiments.

Treatment	Total Number of Oocytes	Number of 2-Cell Embryos	Fertilization Rate (%)	Inhibition (%)
Control	98	78	80 ± 1.7	-
Vehicle	108	96	88 ± 2.0	-
E260	121	27	22 ± 2.4	75

## Data Availability

The original contributions presented in this study are included in the article. Further inquiries can be directed to the corresponding author.

## References

[1] Yanagimachi R. (1994). Fertility of mammalian spermatozoa: Its development and relativity. Zygote.

[2] Ackermann F., Zitranski N., Borth H., Buech T., Gudermann T., Boekhoff I. (2009). CaMKIIalpha interacts with multi-PDZ domain protein MUPP1 in spermatozoa and prevents spontaneous acrosomal exocytosis. J. Cell Sci..

[3] Breitbart H., Grinshtein E. (2023). Mechanisms That Protect Mammalian Sperm from the Spontaneous Acrosome Reaction. Int. J. Mol. Sci..

[4] Sanchez-Cardenas C., Servin-Vences M.R., Jose O., Trevino C.L., Hernandez-Cruz A., Darszon A. (2014). Acrosome reaction and Ca^2+^ imaging in single human spermatozoa: New regulatory roles of [Ca^2+^]_i_. Biol. Reprod..

[5] Mata-Martinez E., Darszon A., Trevino C.L. (2018). pH-dependent Ca^2+^ oscillations prevent untimely acrosome reaction in human sperm. Biochem. Biophys. Res. Commun..

[6] Mata-Martinez E., Sanchez-Cardenas C., Chavez J.C., Guerrero A., Trevino C.L., Corkidi G., Montoya F., Hernandez-Herrera P., Buffone M.G., Balestrini P.A. (2021). Role of calcium oscillations in sperm physiology. Biosystems.

[7] Itoh T., Hasegawa J., Tsujita K., Kanaho Y., Takenawa T. (2009). The tyrosine kinase Fer is a downstream target of the PLD-PA pathway that regulates cell migration. Sci. Signal..

[8] Grinshtain E., Shpungin S., Baum M., Nir U., Breitbart H. (2022). The Fer tyrosine kinase protects sperm from spontaneous acrosome reaction. Dev. Biol..

[9] Elkis Y., Cohen M., Yaffe E., Satmary-Tusk S., Feldman T., Hikri E., Nyska A., Feiglin A., Ofran Y., Shpungin S. (2017). A novel Fer/FerT targeting compound selectively evokes metabolic stress and necrotic death in malignant cells. Nat. Commun..

[10] Schrier I., Slotki-Itzchakov O., Elkis Y., Most-Menachem N., Adato O., Fitoussi-Allouche D., Shpungin S., Unger R., Nir U. (2024). Fer governs mTORC1 regulating pathways and sustains viability of pancreatic ductal adenocarcinoma cells. Front. Oncol..

[11] Liu J., Han Y., Lu T., Yuan D., Lu K., Cai Y., Zhou X., Wang X. (2025). Exosome-transported FER inhibitor suppresses progression of diffuse large B-cell lymphoma via regulating AJUBA/Hippo axis. npj Precis. Oncol..

[12] O’Mara M., Zhang S., Knaus U.G. (2025). Spatiotemporal H(2)O(2) flashes coordinate actin cytoskeletal remodeling and regulate cell migration and wound healing. Nat. Commun..

[13] Ren D., Xia J. (2010). Calcium signaling through CatSper channels in mammalian fertilization. Physiology.

[14] Biscardi J.S., Maa M.C., Tice D.A., Cox M.E., Leu T.H., Parsons S.J. (1999). c-Src-mediated phosphorylation of the epidermal growth factor receptor on Tyr845 and Tyr1101 is associated with modulation of receptor function. J. Biol. Chem..

[15] Etkovitz N., Tirosh Y., Chazan R., Jaldety Y., Daniel L., Rubinstein S., Breitbart H. (2009). Bovine sperm acrosome reaction induced by G-protein-coupled receptor agonists is mediated by epidermal growth factor receptor transactivation. Dev. Biol..

[16] Yaffe E., Hikri E., Elkis Y., Cohen O., Segal A., Makovski A., Varvak A., Shpungin S., Nir U. (2014). Oncogenic properties of a spermatogenic meiotic variant of fer kinase expressed in somatic cells. Cancer Res..

[17] Alvau A., Battistone M.A., Gervasi M.G., Navarrete F.A., Xu X., Sanchez-Cardenas C., De la Vega-Beltran J.L., Da Ros V.G., Greer P.A., Darszon A. (2016). The tyrosine kinase FER is responsible for the capacitation-associated increase in tyrosine phosphorylation in murine sperm. Development.

[18] Wiser A., Sachar S., Ghetler Y., Shulman A., Breitbart H. (2014). Assessment of sperm hyperactivated motility and acrosome reaction can discriminate the use of spermatozoa for conventional in vitro fertilisation or intracytoplasmic sperm injection: Preliminary results. Andrologia.

[19] Visconti P.E. (2009). Understanding the molecular basis of sperm capacitation through kinase design. Proc. Natl. Acad. Sci. USA.

[20] Visconti P.E., Bailey J.L., Moore G.D., Pan D., Olds-Clarke P., Kopf G.S. (1995). Capacitation of mouse spermatozoa. I. Correlation between the capacitation state and protein tyrosine phosphorylation. Development.

[21] Craig A.W., Zirngibl R., Williams K., Cole L.A., Greer P.A. (2001). Mice devoid of fer protein-tyrosine kinase activity are viable and fertile but display reduced cortactin phosphorylation. Mol. Cell Biol..

[22] Kim L., Wong T.W. (1998). Growth factor-dependent phosphorylation of the actin-binding protein cortactin is mediated by the cytoplasmic tyrosine kinase FER. J. Biol. Chem..

[23] Uruno T., Liu J., Zhang P., Fan Y., Egile C., Li R., Mueller S.C., Zhan X. (2001). Activation of Arp2/3 complex-mediated actin polymerization by cortactin. Nat. Cell Biol..

[24] Weaver A.M., Karginov A.V., Kinley A.W., Weed S.A., Li Y., Parsons J.T., Cooper J.A. (2001). Cortactin promotes and stabilizes Arp2/3-induced actin filament network formation. Curr. Biol..

[25] Alekhina O., Burstein E., Billadeau D.D. (2017). Cellular functions of WASP family proteins at a glance. J. Cell Sci..

[26] Pollard T.D. (2007). Regulation of actin filament assembly by Arp2/3 complex and formins. Annu. Rev. Biophys. Biomol. Struct..

[27] Welch M.D., Mullins R.D. (2002). Cellular control of actin nucleation. Annu. Rev. Cell Dev. Biol..

[28] Miyata H., Oura S., Morohoshi A., Shimada K., Mashiko D., Oyama Y., Kaneda Y., Matsumura T., Abbasi F., Ikawa M. (2021). SPATA33 localizes calcineurin to the mitochondria and regulates sperm motility in mice. Proc. Natl. Acad. Sci. USA.

[29] Breitbart H., Etkovitz N. (2011). Role and regulation of EGFR in actin remodeling in sperm capacitation and the acrosome reaction. Asian J. Androl..

[30] Bradford M.M. (1976). A rapid and sensitive method for the quantitation of microgram quantities of protein utilizing the principle of protein-dye binding. Anal. Biochem..

[31] Smith P.K., Krohn R.I., Hermanson G.T., Mallia A.K., Gartner F.H., Provenzano M.D., Fujimoto E.K., Goeke N.M., Olson B.J., Klenk D.C. (1985). Measurement of protein using bicinchoninic acid. Anal. Biochem..

[32] Itach S.B., Finklestein M., Etkovitz N., Breitbart H. (2012). Hyper-activated motility in sperm capacitation is mediated by phospholipase D-dependent actin polymerization. Dev. Biol..

[33] de Oliveira R.V., Dogan S., Belser L.E., Kaya A., Topper E., Moura A., Thibaudeau G., Memili E. (2013). Molecular morphology and function of bull spermatozoa linked to histones and associated with fertility. Reproduction.

